# A Potential Role of the CD47/SIRPalpha Axis in COVID-19 Pathogenesis

**DOI:** 10.3390/cimb43030086

**Published:** 2021-09-22

**Authors:** Katie-May McLaughlin, Denisa Bojkova, Joshua D. Kandler, Marco Bechtel, Philipp Reus, Trang Le, Florian Rothweiler, Julian U. G. Wagner, Andreas Weigert, Sandra Ciesek, Mark N. Wass, Martin Michaelis, Jindrich Cinatl

**Affiliations:** 1 School of Biosciences, University of Kent, Canterbury CT2 7NZ, UK; km625@kent.ac.uk; 2 Institute for Medical Virology, University Hospital, Goethe University Frankfurt am Main, 60596 Frankfurt am Main, Germany; Denisa.Bojkova@kgu.de (D.B.); Joshua.Kandler@kgu.de (J.D.K.); Marco.Bechtel@kgu.de (M.B.); philipp.reus@kgu.de (P.R.); letrang1211@gmail.com (T.L.); f.rothweiler@kinderkrebsstiftung-frankfurt.de (F.R.); Sandra.ciesek@kgu.de (S.C.); 3 Institute for Cardiovascular Regeneration, Goethe University, Theodor Stern Kai 7, 60590 Frankfurt am Main, Germany; j.wagner@med.uni-frankfurt.de; 4 German Center for Cardiovascular Research (DZHK), 60590 Frankfurt am Main, Germany; 5 Faculty for Biological Sciences, Goethe University, 60438 Frankfurt am Main, Germany; 6 Institute of Biochemistry I, Faculty of Medicine, Goethe-University, 60590 Frankfurt am Main, Germany; weigert@biochem.uni-frankfurt.de; 7 German Center for Infection Research, DZIF, External Partner Site, 60590 Frankfurt am Main, Germany; 8 Fraunhofer Institute for Molecular Biology and Applied Ecology (IME), Branch Translational Medicine und Pharmacology, 60590 Frankfurt am Main, Germany

**Keywords:** SARS-CoV-2, COVID-19, antiviral therapy, coronavirus, IAP, CD47, SIRPalpha

## Abstract

The coronavirus SARS-CoV-2 is the cause of the ongoing COVID-19 pandemic. Most SARS-CoV-2 infections are mild or even asymptomatic. However, a small fraction of infected individuals develops severe, life-threatening disease, which is caused by an uncontrolled immune response resulting in hyperinflammation. However, the factors predisposing individuals to severe disease remain poorly understood. Here, we show that levels of CD47, which is known to mediate immune escape in cancer and virus-infected cells, are elevated in SARS-CoV-2-infected Caco-2 cells, Calu-3 cells, and air−liquid interface cultures of primary human bronchial epithelial cells. Moreover, SARS-CoV-2 infection increases SIRPalpha levels, the binding partner of CD47, on primary human monocytes. Systematic literature searches further indicated that known risk factors such as older age and diabetes are associated with increased CD47 levels. High CD47 levels contribute to vascular disease, vasoconstriction, and hypertension, conditions that may predispose SARS-CoV-2-infected individuals to COVID-19-related complications such as pulmonary hypertension, lung fibrosis, myocardial injury, stroke, and acute kidney injury. Hence, age-related and virus-induced CD47 expression is a candidate mechanism potentially contributing to severe COVID-19, as well as a therapeutic target, which may be addressed by antibodies and small molecules. Further research will be needed to investigate the potential involvement of CD47 and SIRPalpha in COVID-19 pathology. Our data should encourage other research groups to consider the potential relevance of the CD47/ SIRPalpha axis in their COVID-19 research.

## 1. Introduction

Severe acute respiratory syndrome coronavirus 2 (SARS-CoV-2) is causing the ongoing coronavirus disease 2019 (COVID-19) outbreak [1,2], which has resulted in more than 210 million confirmed cases and more than 4.4 million confirmed COVID-19-associated deaths so far [3]. Older age; being male; and conditions such as diabetes, hypertension, and obesity are associated with an increased risk of severe COVID-19 [1,4].

The first COVID-19 vaccines have been developed [2], and their roll-out has started in many countries. However, it will take a significant time until large parts of the global population will be vaccinated, and there is growing concern about the emergence of escape variants that can bypass the immunity conferred by the current vaccines and previous SARS-CoV-2 infections [5,6,7,8,9,10]. Thus, for the foreseeable future, there will be a need for improved COVID-19 therapies.

Currently, the therapeutic options for COVID-19 are still very limited [2,11]. COVID-19 therapies can either directly inhibit SARS-CoV-2 replication or target other COVID-19-associated pathophysiological processes, such as corticosteroids, which are anticipated to control COVID-19-related cytokine storm and hyperinflammation [12]. Dexamethasone and potentially other corticosteroids increase survival in patients who depend on oxygen support [13,14]. In a controlled open-label trial, dexamethasone reduced mortality in patients receiving oxygen with (from 41.1% to 29.3%) or without (from 26.2% to 23.3%) mechanical ventilation, but increased mortality in patients not requiring oxygen support [13]. Other immunomodulatory therapy candidates are being tested, but conclusive results are pending [11]. Further COVID-19 therapeutics under investigations include anticoagulants that target COVID-19-induced systemic coagulation and thrombosis (coagulopathy) [15].

However, it would be much better to have effective antiviral treatments that reliably prevent COVID-19 disease progression to a stage where immunomodulators and anticoagulants are needed. The antiviral drug remdesivir was initially described to reduce the recovery time from 15 to 10 days, and the 29-day mortality from 15.2% to 11.4% [11,16]. However, other trials did not confirm this and conclusive evidence on the efficacy of remdesivir remains to be established [11]. The JAK inhibitor baricitinib, which interferes with cytokine signaling, was reported to improve therapy outcomes in combination with remdesivir in a double-blind, randomized, placebo-controlled trial, in which patients were either treated with remdesivir plus baricitinib or remdesivir plus placebo [17]. Moreover, convalescent sera and monoclonal antibodies are under clinical investigation for COVID-19 treatment [18,19,20].

Ideally, antiviral therapies are used early in the disease course to prevent disease progression to the later immunopathology-driven stages [21]. However, only a small proportion of patients develop severe disease [21]. Therefore, a better understanding of the underlying processes is required to identify the patients who could develop severe disease as early as possible.

CD47 is the receptor of thrombospondin-1 (THBS1) and the counter-receptor for signal regulatory protein-α (SIRPα). CD47 interaction with SIRPα inhibits the activation of macrophages and dendritic cells, and thrombospondin-1/ CD47 signaling inhibits T cell activation [22,23]. Cellular surface levels of CD47 modulate immune responses in infectious diseases caused by parasites, bacteria, and viruses [22]. Typically, high CD47 levels prevent the immune recognition of virus-infected cells [22,24]. Moreover, cancer cells have been described to avoid immune recognition by upregulating CD47 [22]. Here, we investigated the potential role of the ubiquitously expressed cell surface glycoprotein CD47 in severe COVID-19.

## 2. Materials and Methods

### 2.1. Cell Culture

Calu-3 cells (ATCC, #HTB-55) were grown at 37 °C in minimal essential medium (MEM) supplemented with 10% fetal bovine serum (FBS), 100 IU/mL penicillin, and 100 μg/mL of streptomycin. All of the culture reagents were purchased from Sigma. The cells were regularly authenticated by short tandem repeat (STR) analysis and were tested for mycoplasma contamination.

Primary human bronchial epithelial cells were purchased from ScienceCell. For differentiation to air−liquid interface (ALI) cultures, the cells were thawed and passaged once in PneumaCult-Ex Medium (StemCell Technologies, Vancouver, BC, Kanada) and then seeded on transwell inserts (12-well plate, Sarstedt, Nümbrecht, Germany) at 4 × 10^4^ cells/insert. Once the cell layers reached confluency, the medium on the apical side of the transwell was removed, and the medium in the basal chamber was replaced with PneumaCult ALI Maintenance Medium (StemCell Technologies, Vancouver, BC, Kanada) including an antibiotic/antimycotic solution (Sigma Aldrich, Saint Louis, MO, USA) and MycoZap Plus PR (Lonza, Basel, Switzerland). Over a period of four weeks, the medium was changed and cell layers were washed with PBS every other day. Criteria for successful differentiation were the development of ciliated cells and ciliary movement, an increase in transepithelial electric resistance indicative of the formation of tight junctions, and mucus production.

Human monocytes were isolated from the buffy coats of healthy donors (RK-Blutspendedienst Baden-Württemberg-Hessen, Institut für Transfusionsmedizin und Immunhämatologie Frankfurt am Main, Germany). After centrifugation on a Ficoll (Pancoll, PAN-Biotech, Aidenbach, Germany) density gradient, mononuclear cells were collected from the interface, washed with PBS, and plated on cell culture dishes (Cell+, Saarstedt, Nümbrecht, Germany) in RPMI1640 (Gibco, ThermoFisher Scientific, Waltham, MA, USA) supplemented with 100 IU/mL penicillin and 100 μg/mL streptomycin. After incubation for 90 min (37 °C, 5% CO_2_), non-adherent cells were removed, and the medium was changed to RPMI1640 supplemented with 100 IU/mL penicillin, 100 μg/mL of streptomycin, and 3% human serum (RK-Blutspendedienst Baden-Württemberg-Hessen, Institut für Transfusionsmedizin und Immunhämatologie Frankfurt am Main, Germany).

### 2.2. Virus Infection

SARS-CoV-2/7/Human/2020/Frankfurt (SARS-CoV-2/FFM7) was isolated and cultivated in Caco2 cells (DSMZ, #AC169), as previously described [25,26]. Virus titers were determined as TCID50/mL in confluent cells in 96-well microtiter plates [27,28].

Monocytes were infected at an MOI of 1 with SARS-CoV-2/FFM7 for 2 h. After infection, the cells were washed three times with PBS and subsequently cultivated in RPMI1640 (Gibco) supplemented with 100 IU/mL penicillin and 100 μg/mL streptomycin.

### 2.3. Western Blot

The cells were lysed using a Triton-X-100 sample buffer, and the proteins were separated by SDS-PAGE. Detection occurred using specific antibodies against CD47 (1:100 dilution, CD47 Antibody, anti-human, Biotin, REAfinity™, # 130-101-343, Miltenyi Biotec, Bergisch Gladbach, Germany), SARS-CoV-2 N (1:1000 dilution, SARS-CoV-2 Nucleocapsid Antibody, Rabbit MAb, #40143-R019, Sino Biological, Beijing, China), SIRPα (1:1000 dilution, SIRPα/SHPS1 (D6I3M) Rabbit mAb #13379, Cell Signaling, Danvers, MA, USA), and GAPDH (1:1000 dilution, Anti-G3PDH Human Polyclonal Antibody, #2275-PC-100, Trevigen, Gaithersburg, MD, USA). Protein bands were visualized and quantified by laser-induced fluorescence using an infrared scanner for protein quantification (Odyssey, Li-Cor Biosciences, Bad Homburg vor der Höhe, Germany).

### 2.4. qPCR

SARS-CoV-2 RNA from cell culture supernatant samples was isolated using the AVL buffer and QIAamp Viral RNA Kit (Qiagen, Vienna, Austria) according to the manufacturer’s instructions. SARS-CoV-2 RNA from the cell lysates was isolated using an RTL Buffer and the RNeasy Mini Kit (Qiagen, Vienna, Austria), according to the manufacturer’s instructions. Absorbance-based quantification of the RNA yield was performed using the Genesys 10S UV−ViIS Spectrophotometer (Thermo Scientific, Waltham, MA, USA). RNA was subjected to OneStep qRT-PCR analysis using the Luna Universal One-Step RT-qPCR Kit (New England Biolabs, Hitchin, UK) and a CFX96 Real-Time System, C1000 Touch Thermal Cycler. Primers were adapted from the WHO protocol29 targeting the open reading frame for RNA-dependent RNA polymerase (RdRp): RdRP_SARSr-F2 (GTG ARA TGG TCA TGT GTG GCG G) and RdRP_SARSr-R1 (CAR ATG TTA AAS ACA CTA TTA GCA TA) using 0.4 µM per reaction. Standard curves were created using plasmid DNA (pEX-A128-RdRP) harboring the corresponding amplicon regions for the RdRP target sequence according to GenBank accession number NC_045512. For each condition three biological replicates were used. The mean and standard deviation were calculated for each group.

### 2.5. Data Acquisition and Analysis

Normalized protein abundance data from SARS-CoV-2-infected Caco-2 cells were derived from a recent publication [29] and are available from the PRIDE repository [30] (dataset identifier PXD017710). Data were subsequently normalized using summed intensity normalization for sample loading, followed by internal reference scaling and trimmed mean of M normalization. Mean protein abundance was plotted using the function *ggdotplot* of the R package ggpubr. *p*-values were determined by two-sided Student’s *t*-test.

Raw read counts from SARS-CoV-2-infected Calu-3 cells were derived from a recent publication [31] via the Gene Expression Omnibus (GEO) database (accession: GSE147507) and were processed using DESeq2. Normalized gene counts were plotted using the function *ggdotplot* of the R package ggpubr. *p*-values were determined by two-sided Student’s *t*-test.

### 2.6. Literature Review

Relevant articles were identified by using the search terms “CD47 aging”, “CD47 hypertension”, “CD47 diabetes”, and “CD47 obesity” in PubMed (https://pubmed.ncbi.nlm.nih.gov accessed on 17 February 2021) on the basis of the principles outlined in the PRISMA guidelines (http://prisma-statement.org accessed on 17 February 2021). Articles in English were included in the analysis when they contained original data on the influence of aging, diabetes, diabetes, or obesity on CD47 expression levels and/or the relevance of CD47 with regard to pathological conditions observed in severe COVID-19. Two reviewers independently analyzed the articles for relevant information and then agreed a list of relevant articles.

## 3. Results

### 3.1. SARS-CoV-2 Infection Results in Enhanced CD47 Expression

**Figure 1:**
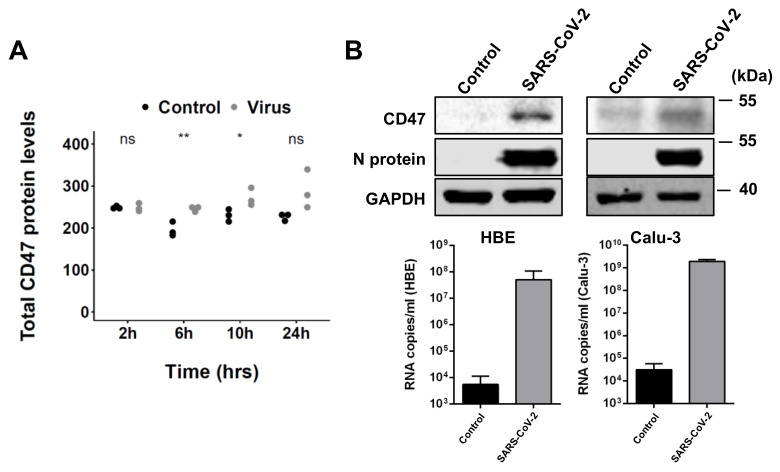
SARS-CoV-2 infection is associated with increased CD47 levels. (**A**) TF protein abundance in uninfected (control) and SARS-CoV-2-infected (virus) Caco-2 cells (normalized signal intensity, data derived from [23]). *p*-values were determined using two-sided Student’s *t*-test. (**B**) CD47 and SARS-CoV-2 N protein levels and virus titers (genomic RNA determined by PCR) in SARS-CoV-2 strain FFM7 (MOI 1)-infected air−liquid interface cultures of primary human bronchial epithelial (HBE) cells and SARS-CoV-2 strain FFM7 (MOI 0.1)-infected Calu-3 cells. Uncropped blots are provided in Figure S1. *p*-values were determined by two-sided Student’s *t*-test. * *p* < 0.05, ** *p* < 0.01.

A publicly available proteomics dataset [29] indicated an increased CD47 expression in SARS-CoV-2-infected Caco2 colorectal carcinoma cells (Figure 1A). We also detected enhanced CD47 levels in SARS-CoV-2-infected primary human bronchial epithelial cells (HBE) grown in air−liquid interface (ALI) cultures [32] and Calu-3 lung cancer cells (Figure 1B and Figure S1). An analysis of the transcriptomics data from another study also indicated increased CD47 levels in SARS-CoV-2-infected Calu-3 cells (Figure S2) [31]. Flow cytometry analysis confirmed increased CD47 levels in SARS-CoV-2-infected Caco2 cells (Figure S3).

### 3.2. Increased SIRPα Levels in SARS-CoV-2-Infected Monocytes

**Figure 2:**
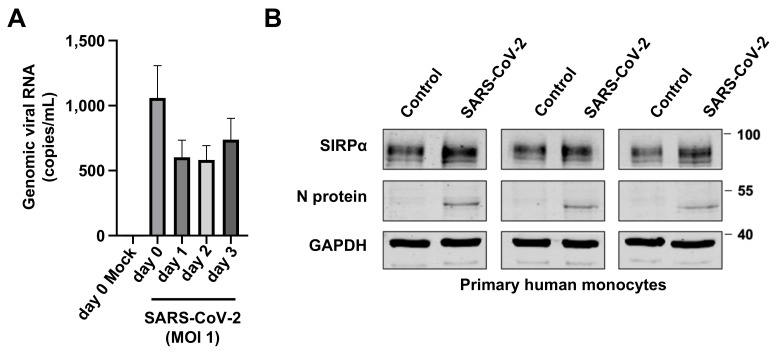
SARS-CoV-2 infection increases SIRPα in primary human monocytes. (**A**) SARS-CoV-2. The (MOI 1) infection of primary human monocytes does not result in the production of genomic viral RNA, as detected by PCR. (**B**) SARS-CoV-2 strain FFM7 (MOI 1)-infected primary human monocytes display enhanced SIRPα levels. Uncropped blots are provided in Figure S4. Quantification of the protein levels is provided in Figure S5.

CD47 inhibits the activity of innate immune cells via the interaction with SIRPα [22,23]. Hence, we next investigated whether the SARS-CoV-2 infection of monocytes may impact SIRPα levels. SARS-CoV-2 did not result in a productive infection of primary human monocytes as indicated by a lack of an increase in genomic RNA levels (Figure 2A). However, SARS-CoV-2 infection resulted in increased SIRPα levels in primary monocytes (Figure 2B, Figures S4 and S5). Hence, SARS-CoV-2 may interfere with both players of the CD47/SIRPα axis.

### 3.3. CD47 and COVID-19 Risk Factors

To further investigate the potential role of CD47 in severe COVID-19, we performed systematic literature searches on the relationship of CD47 and the known COVID-19 risk factors of “ageing”, “diabetes”, and “obesity”.

#### 3.3.1. CD47 and Aging

**Figure 3:**
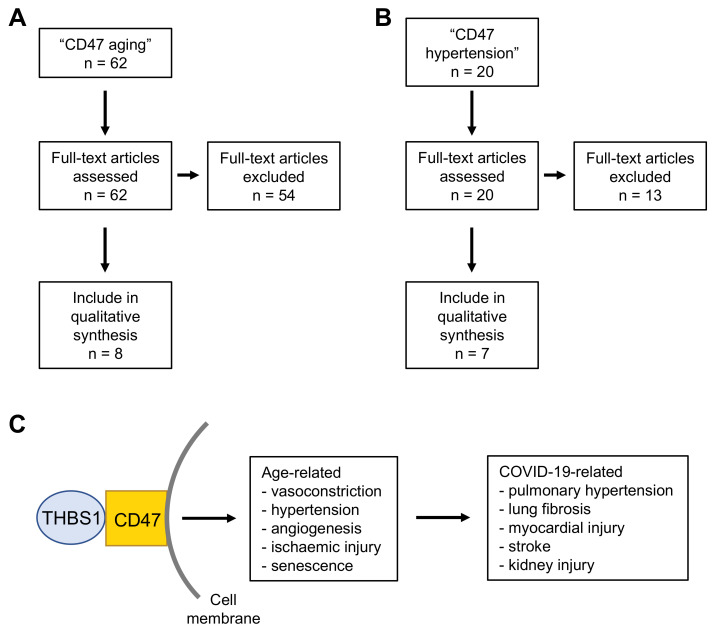
Results of the PubMed (https://pubmed.ncbi.nlm.nih.gov accessed on 17 February 2021) literature search for “CD47 aging” (**A**) and “CD47 hypertension” (**B**). (**C**) Overview figure of the data derived from the literature searches. Age-related increased CD47 levels may contribute to pathogenic conditions associated with severe COVID-19.

The risk of severe COVID-19 disease and COVID-19 death increases with age [1]. A literature search in PubMed (https://pubmed.ncbi.nlm.nih.gov, accessed on 17 February 2020) using the terms “CD47” and “aging” resulted in 62 hits (Table S1). Eight of these articles contained information that supports a link between age-related increased CD47 levels and an elevated risk of severe COVID-19 (Figure 3 and Table S1). One article suggested that alpha-tocopherol reduced age-associated streptococcus pneumoniae lung infection in mice through CD47 downregulation [33], which is in accordance with the known immunosuppressive functions of CD47 [22,23].

The remaining seven articles reported on age-related increased CD47 levels in vascular cells that are associated with reduced vasodilatation and blood flow (Table S1), as CD47 signaling inhibits NO-mediated activation of soluble guanylate cyclase and in turn vasodilatation [34,35]. As reduced vasodilatation can cause hypertension [36], we performed a follow-up literature search using the search terms “CD47 hypertension” (Table S2). This resulted in 20 hits, including a further six relevant studies (Figure 3B and Table S2). The evidence supporting a link between aging and/or hypertension and increased CD47 levels is summarized in Table 1.

Initial experiments showed that loss or inhibition of CD47 prevented age- and diet-induced vasculopathy and reduced damage caused by ischemic injury in mice [37]. CD47-deficient mice indicated that CD47 functions as a vasopressor, and the mice were also shown to be leaner and to display an enhanced physical performance and a more efficient metabolism [38,39]. In agreement, CD47 was upregulated in clinical pulmonary hypertension and contributed to pulmonary arterial vasculopathy and dysfunction in mouse models [40,41]. Age-related increased CD47 levels further affected peripheral blood flow and wound healing in mice [42] and NO-mediated vasodilatation of coronary arterioles of rats [43]. Moreover, thrombospondin-1/CD47 signaling was shown to induce ageing-associated senescence in endothelial cells [44,45] and age-associated deterioration in angiogenesis, blood flow, and glucose homeostasis [46].

Increased CD47 levels were also detected in the lungs of a sickle cell disease patient with pulmonary arterial hypertension, and vasculopathy and pulmonary hypertension were reduced in a CD47-null mouse model of sickle cell disease [47,48]. Finally, anti-CD47 antibodies reversed fibrosis in various organs in mouse models [49], which may be relevant in the context of COVID-19-associated pulmonary fibrosis [50].

In addition to their immunosuppressive activity, ageing-related increased CD47 levels may thus be involved in vascular disease, vasoconstriction, and hypertension, and predispose COVID-19 patients to related pathologies such as pulmonary hypertension, lung fibrosis, myocardial injury, stroke, and acute kidney injury [4,50,51,52,53,54,55,56,57].

#### 3.3.2. CD47 and Diabetes

**Figure 4:**
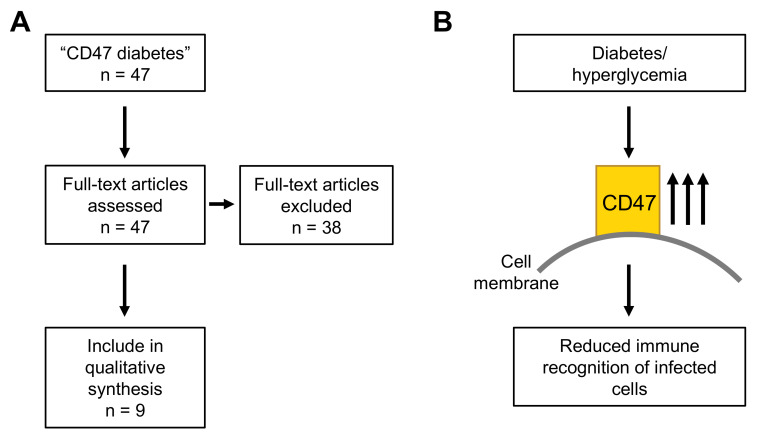
Results of the PubMed (https://pubmed.ncbi.nlm.nih.gov accessed on 17 February 2021) literature search for “CD47 diabetes” (**A**). (**B**) Overview figure of the data derived from the literature search. Hyperglycemia- and diabetes-induced increased CD47 levels may contribute to the immune escape of SARS-CoV-2-infected cells.

Diabetes has been associated with an increased risk of severe COVID-19 and COVID-19-related death [4]. A PubMed search for “CD47 diabetes” produced 47 hits, nine of which reported increased CD47 levels in response to hyperglycemia and/or diabetes (Figure 4A and Table S3).

Hyperglycemia protected CD47 from cleavage, resulting in increased CD47 levels [58,59,60,61]. In agreement, increased CD47 levels were detected in various cell types and tissues in rat diabetes models and diabetes patients [62,63,64,65,66] (Table 2). Therefore, diabetes-induced increased CD47 levels may interfere with the recognition of SARS-CoV-2-infected cells by the immune system [22,23] (Figure 4B).

#### 3.3.3. CD47 and Obesity

As obesity is another risk factor for severe COVID-19 [4], we also performed a PubMed search for “CD47 obesity”, which resulted in eight hits, two of which provided potentially relevant information (Table S4). The results indicated that CD47-deficient mice were leaner, probably as a consequence of elevated lipolysis [67,68]. Hence, low CD47 levels may be associated both with lower weight and increased immune recognition of virus-infected cells [22,23,67,68], but there is no direct evidence suggesting that obesity may also directly increase CD47 expression. However, obesity may at least indirectly contribute to enhanced CD47 levels as a risk factor for diabetes and hypertension [4].

## 4. Discussion

Here, we show that the levels of CD47 are elevated in SARS-CoV-2-infected Caco-2 cells, Calu-3 cells, and air−liquid interface cultures of primary human bronchial epithelial cells. CD47 exerts an immunosuppressive activity via interaction with SIRPα in immune cells and as a thrombospondin-1 receptor [22,23]. In this context, human CD47 expression is discussed as a strategy to enable the xenotransplantation of organs from pigs to humans [69,70]. Moreover, a high CD47 expression is an immune escape mechanism observed in cancer cells, and anti-CD47 antibodies are under investigation as cancer immunotherapeutics [23,71]. Due its immunosuppressive action, CD47 expression is also discussed as a target for the treatment of viral and bacterial pathogens, including SARS-CoV-2 [22,24,72]. It has been demonstrated that cells infected with different viruses display enhanced CD47 levels, which function as a “do not eat me” signal, which interferes with the immune recognition of virus-infected cells [24]. Thus, our data indicating increased CD47 levels in a range of SARS-CoV-2 infection models and clinical samples further support the potential role of CD47 as a drug target for the mediation of a more effective antiviral immune response.

Moreover, we found that, although SARS-CoV-2 did not replicate in primary human monocytes, it increased the levels of the CD47 binding partner SIRPα in these cells. Hence, SARS-CoV-2 infection may affect the immune recognition of SARS-CoV-2-infected cells by upregulating both players of the CD47/SIRPα axis. Notably, other viruses and bacteria have previously been described to increase host cell SIRPα levels [73,74]. Moreover, elevated SIRP-α expression was recently reported in blood mononuclear cells of COVID-19 patients [75], and the CD47/SIRP-α interaction was associated with lung damage in severe COVID-19 [76].

High SARS-CoV-2 loads are associated with more severe COVID-19 and a higher risk of patient death [77,78]. Therefore, high CD47 and/or SIRPα levels may affect initial virus control resulting in enhanced virus levels, which may eventually lead to the hyperinflammation and immunopathology observed in severe COVID-19. Moreover, innate immune responses appear to be critically involved in the early control of SARS-CoV-2, and the deregulation of monocytes and macrophages seems to be a factor contributing to severe COVID-19 [79,80].

Older age, diabetes, and obesity are known risk factors for COVID-19 morbidity and mortality [1,4]. Hence, we performed a series of systematic reviews to identify potential connections between CD47 and these processes. The results indicated an ageing-related increase in CD47 expression, which may contribute to the increased COVID-19 vulnerability in older patients [1]. Moreover, high CD47 levels are known to be involved in vascular disease, vasoconstriction, and hypertension, which may predispose SARS-CoV-2-infected individuals to various conditions associated with severe COVID-19 related, including pulmonary hypertension, lung fibrosis, myocardial injury, stroke, and acute kidney injury [4,50,51,52,53,54,55,56,57].

High CD47 levels have also been reported as a consequence of hyperglycemia and diabetes, which may contribute to the high risk of severe COVID-19 in diabetic patients [4]. Although there is no known direct impact of obesity on CD47 levels, obesity is associated with an increased risk of diabetes and other ageing-related conditions such as hypertension, which may result in elevated COVID-19 vulnerability [4].

## 5. Conclusions

Severe COVID-19 disease is a consequence of hyperinflammation (“cytokine storm”) in response to SARS-CoV-2 infection [1]. Hence, the optimal time window for antiviral intervention is as early as possible to prevent disease progression to severe stages driven by immunopathology [1]. As the vast majority of cases are mild or even asymptomatic [1], an improved understanding of the processes underlying severe COVID-19 is required for the early identification of patients at high risk.

Here, we investigated a potential role of CD47 expression in determining COVID-19 severity. SARS-CoV-2 infection resulted in an enhanced expression of CD47 in different cell types. CD47 interferes with the host immune response by mechanisms including binding to SIRPα on immune cells. Notably, SARS-CoV-2 also increased SIRPα levels on primary human monocytes, indicating that SARS-CoV-2 can interfere with the immune response by elevating both binding partners of the CD47/ SIRPα axis.

Moreover, CD47 levels are elevated in groups at high risk for COVID-19, such as older individuals and individuals with hypertension and/or diabetes. Thus, high CD47 levels may predispose these groups to severe COVID-19. Additionally, CD47 is a potential therapeutic target that can be addressed with antibodies and small molecules [22,23,24,72]. Notably, targeting SIRPα also represents a therapeutic option that may be more specific, as SIRPα is restricted to monocytes and macrophages [81]. Further research will be needed to define the roles of CD47 and/or SIRPalpha in COVID-19 in more detail. Thus, our findings should also encourage other research groups to consider the potential relevance of these molecules in their COVID-19 research.

## Figures and Tables

**Table 1:** Evidence supporting a link between aging and/or hypertension and increased CD47 levels.

Reference	Link between Aging and/or Hypertension and Increased CD47 Levels
[33]	CD47 downregulation may be involved in the alpha-tocopherol-mediated inhibition of age-associated streptococcus pneumoniae lung infection in mice
[37]	Blocking thrombospondin-1/CD47 signaling alleviates deleterious effects of aging on tissue responses to ischemia
[38]	CD47 null mice indicate that CD47 functions as a vasopressor
[39]	CD47-null mice are leaner—loss of signaling from the TSP1-CD47 system promotes the accumulation of normally functioning mitochondria in a tissue-specific and age-dependent fashion, leading to enhanced physical performance, lower reactive oxygen species production, and more efficient metabolism
[40]	High CD47 levels promote pulmonary arterial hypertension in the lungs from humans and mice
[41]	TSP1-CD47 signaling is upregulated in clinical pulmonary hypertension and contributes to pulmonary arterial vasculopathy and dysfunction
[42]	Increased THBS1/CD47 signaling contributes to reduced skin blood flow and wound healing in aged mice
[43]	CD47 blocks NO-mediated vasodilatation
[44]	THBS1/CD47 signaling drives endothelial cell senescence
[45]	TSP1 promotes ageing-associated human and mouse endothelial cell senescence through CD47
[46]	Increased CD47 expression causes age-associated deterioration in angiogenesis, blood flow, and glucose homeostasis
[47]	Increased CD47 levels in the lung of a sickle cell disease patient with pulmonary arterial hypertension relative to control tissues
[48]	Pulmonary hypertension reduced in a CD47-null mouse model of sickle cell disease
[49]	Anti-CD47 antibodies reversed fibrosis in various organs in mouse models

**Table 2:** Evidence supporting a link between diabetes and increased CD47 levels.

Reference	Link between Aging and/ or Hypertension and Increased CD47 Levels
[58]	Hyperglycemia protects CD47 from cleavage
[59]	Hyperglycemia protects CD47 from cleavage
[60]	Hyperglycemia protects CD47 from cleavage
[61]	Hyperglycemia protects CD47 from cleavage
[62]	CD47 is involved in pathophysiological changes in retinal cells in response to hyperglycemia in cell cultures and rats
[63]	Elevated CD47 mRNA levels both in the hippocampus and prefrontal cortex of a type-2 diabetes rat model
[64]	Increased levels of CD47 in epiretinal membranes with active neovascularization in proliferative diabetic retinopathy
[65]	Increased THBS1/CD47 signaling in bone marrow-derived angiogenic cells in a rat diabetes model
[66]	Increased diabetes-associated CD47 levels inhibit angiogenesis and wound healing in a diabetes model in rats

## Data Availability

Data were derived from [23] via the PRIDE partner repository with the dataset identifier PXD017710, and from [25] via the Gene Expression Omnibus (GEO) database (accession: GSE147507).

## Supplementary Materials

The following are available online at https://www.mdpi.com/article/10.3390/cimb43030086/s1. Figure S1: Uncropped Western blots to Figure 1. Figure S2: CD47 mRNA levels in SARS-CoV-2-infected Calu-3 cells (data derived from [25]). Figure S3: CD47 levels in SARS-CoV-2 (MOI 0.1)-infected Caco2 cells as determined by flow cytometry; Figure S4: Uncropped Western blots to Figure 2; Figure S5: Quantification of SIRPα levels in SARS-CoV-2-infected primary human monocytes; Table S1: Literature search for “CD47 aging”. Table S2: Literature search for “CD47 hypertension”. Table S3: Literature search for “CD47 diabetes”. Table S4: Literature search for “CD47 obesity”.

## References

[1] Hokello J., Sharma A.L., Shukla G.C., Tyagi M. (2020). A narrative review on the basic and clinical aspects of the novel SARS-CoV-2, the etiologic agent of COVID-19. Ann. Transl. Med..

[2] Chilamakuri R., Agarwal S. (2021). COVID-19: Characteristics and Therapeutics. Cells.

[3] Dong E., Du H., Gardner L. (2020). An interactive web-based dashboard to track COVID-19 in real time. Lancet Infect. Dis..

[4] Shah H., Khan M.S.H., Dhurandhar N.V., Hegde V. (2021). The triumvirate: Why hypertension, obesity, and diabetes are risk factors for adverse effects in patients with COVID-19. Acta Diabetol..

[5] Andreano E., Piccini G., Licastro D., Casalino L., Johnson N.V., Paciello I., Monego S.D., Pantano E., Manganaro N., Manenti A. (2020). SARS-CoV-2 escape in vitro from a highly neutralizing COVID-19 convalescent plasma. bioRxiv.

[6] Kemp S.A., Collier D.A., Datir R., Ferreira I., Gayed S., Jahun A., Hosmillo M., Rees-Spear C., Mlcochova P., Lumb I.U. (2020). Neutralising antibodies in Spike mediated SARS-CoV-2 adaptation. medRxiv.

[7] Liu Z., VanBlargan L.A., Rothlauf P.W., Bloyet L.M., Chen R.E., Stumpf S., Zhao H., Errico J.M., Theel E.S., Ellebedy A.H. (2020). Landscape analysis of escape variants identifies SARS-CoV-2 spike mutations that attenuate monoclonal and serum antibody neutralization. bioRxiv.

[8] Weisblum Y., Schmidt F., Zhang F., DaSilva J., Poston D., Lorenzi J.C., Muecksch F., Rutkowska M., Hoffmann H.H., Michailidis E. (2020). Escape from neutralizing antibodies by SARS-CoV-2 spike protein variants. Elife.

[9] Sabino E.C., Buss L.F., Carvalho M.P.S., Prete C.A., Crispim M.A.E., Fraiji N.A., Pereira R.H.M., Parag K.V., da Silva Peixoto P., Kraemer M.U.G. (2021). Resurgence of COVID-19 in Manaus, Brazil, despite high seroprevalence. Lancet.

[10] Wibmer C.K., Ayres F., Hermanus T., Madzivhandila M., Kgagudi P., Lambson B.E., Vermeulen M., van den Berg K., Rossouw T., Boswell M. (2021). SARS-CoV-2 501Y.V2 escapes neutralization by South African COVID-19 donor plasma. bioRxiv.

[11] Rebold N., Holger D., Alosaimy S., Morrisette T., Rybak M. (2021). COVID-19: Before the Fall, An Evidence-Based Narrative Review of Treatment Options. Infect. Dis. Ther..

[12] Pum A., Ennemoser M., Adage T., Kungl A.J. (2021). Cytokines and Chemokines in SARS-CoV-2 Infections-Therapeutic Strategies Targeting Cytokine Storm. Biomolecules.

[13] Horby P., Lim W.S., Emberson J.R., Mafham M., Bell J.L., Linsell L., Staplin N., Brightling C., Ustianowski A., RECOVERY Collaborative Group (2021). Dexamethasone in Hospitalized Patients with Covid-19—Preliminary Report. N. Engl. J. Med..

[14] Sterne J.A.C., Murthy S., Diaz J.V., Slutsky A.S., Villar J., Angus D.C., Annane D., Azevedo L.C.P., Berwanger O., WHO Rapid Evidence Appraisal for COVID-19 Therapies (REACT) Working Group (2020). Association Between Administration of Systemic Corticosteroids and Mortality Among Critically Ill Patients With COVID-19: A Meta-analysis. JAMA.

[15] Hadid T., Kafri Z., Al-Katib A. (2020). Coagulation and anticoagulation in COVID-19. Blood Rev..

[16] Beigel J.H., Tomashek K.M., Dodd L.E., Mehta A.K., Zingman B.S., Kalil A.C., Hohmann E., Chu H.Y., Luetkemeyer A., Kline S. (2020). Remdesivir for the Treatment of Covid-19—Final Report. N. Engl. J. Med..

[17] Kalil A.C., Patterson T.F., Mehta A.K., Tomashek K.M., Wolfe C.R., Ghazaryan V., Marconi V.C., Ruiz-Palacios G.M., Hsieh L., Kline S. (2021). Baricitinib plus Remdesivir for Hospitalized Adults with Covid-19. N. Engl. J. Med..

[18] Tuccori M., Ferraro S., Convertino I., Cappello E., Valdiserra G., Blandizzi C., Maggi F., Focosi D. (2020). Anti-SARS-CoV-2 neutralizing monoclonal antibodies: Clinical pipeline. MAbs.

[19] Devarasetti P.K., Rajasekhar L., Baisya R., Sreejitha K.S., Vardhan Y.K. (2021). A review of COVID-19 convalescent plasma use in COVID-19 with focus on proof of efficacy. Immunol. Res..

[20] Weinreich D.M., Sivapalasingam S., Norton T., Ali S., Gao H., Bhore R., Musser B.J., Soo Y., Rofail D., Im J. (2021). REGN-COV2, a Neutralizing Antibody Cocktail, in Outpatients with Covid-19. N. Engl. J. Med..

[21] Salzberger B., Buder F., Lampl B., Ehrenstein B., Hitzenbichler F., Holzmann T., Schmidt B., Hanses F. (2020). Epidemiology of SARS-CoV-2. Infection.

[22] Cham L.B., Adomati T., Li F., Ali M., Lang K.S. (2020). CD47 as a Potential Target to Therapy for Infectious Diseases. Antibodies.

[23] Kaur S., Cicalese K.V., Bannerjee R., Roberts D.D. (2020). Preclinical and Clinical Development of Therapeutic Antibodies Targeting Functions of CD47 in the Tumor Microenvironment. Antib. Ther..

[24] Tal M.C., Torrez Dulgeroff L.B., Myers L., Cham L.B., Mayer-Barber K.D., Bohrer A.C., Castro E., Yiu Y.Y., Lopez Angel C., Pham E. (2020). Upregulation of CD47 Is a Host Checkpoint Response to Pathogen Recognition. mBio.

[25] Hoehl S., Berger A., Kortenbusch M., Cinatl J., Bojkova D., Rabenau H., Behrens P., Böddinghaus B., Götsch U., Naujoks F. (2020). Evidence of SARS-CoV-2 Infection in Returning Travelers from Wuhan, China. N. Engl. J. Med..

[26] Toptan T., Hoehl S., Westhaus S., Bojkova D., Berger A., Rotter B., Hoffmeier K., Cinatl J., Ciesek S., Widera M. (2020). Optimized qRT-PCR Approach for the Detection of Intra- and Extra-Cellular SARS-CoV-2 RNAs. Int. J. Mol. Sci..

[27] Cinatl J., Morgenstern B., Bauer G., Chandra P., Rabenau H., Doerr H.W. (2003). Glycyrrhizin, an active component of liquorice roots, and replication of SARS-associated coronavirus. Lancet.

[28] Cinatl J., Michaelis M., Morgenstern B., Doerr H.W. (2005). High-dose hydrocortisone reduces expression of the pro-inflammatory chemokines CXCL8 and CXCL10 in SARS coronavirus-infected intestinal cells. Int. J. Mol. Med..

[29] Bojkova D., Klann K., Koch B., Widera M., Krause D., Ciesek S., Cinatl J., Münch C. (2020). Proteomics of SARS-CoV-2-infected host cells reveals therapy targets. Nature.

[30] Perez-Riverol Y., Csordas A., Bai J., Bernal-Llinares M., Hewapathirana S., Kundu D.J., Inuganti A., Griss J., Mayer G., Eisenacher M. (2019). The PRIDE database and related tools and resources in 2019: Improving support for quantification data. Nucleic Acids Res..

[31] Blanco-Melo D., Nilsson-Payant B.E., Liu W.C., Uhl S., Hoagland D., Møller R., Jordan T.X., Oishi K., Panis M., Sachs D. (2020). Imbalanced Host Response to SARS-CoV-2 Drives Development of COVID-19. Cell.

[32] Bojkova D., Bechtel M., McLaughlin K.M., McGreig J.E., Klann K., Bellinghausen C., Rohde G., Jonigk D., Braubach P., Ciesek S. (2020). Aprotinin Inhibits SARS-CoV-2 Replication. Cells.

[33] Bou Ghanem E.N., Clark S., Du X., Wu D., Camilli A., Leong J.M., Meydani S.N. (2015). The α-tocopherol form of vitamin E reverses age-associated susceptibility to streptococcus pneumoniae lung infection by modulating pulmonary neutrophil recruitment. J. Immunol..

[34] Isenberg J.S., Frazier W.A., Roberts D.D. (2008). Thrombospondin-1: A physiological regulator of nitric oxide signaling. Cell. Mol. Life Sci..

[35] Miller T.W., Isenberg J.S., Roberts D.D. (2010). Thrombospondin-1 is an inhibitor of pharmacological activation of soluble guanylate cyclase. Br. J. Pharmacol..

[36] Touyz R.M., Alves-Lopes R., Rios F.J., Camargo L.L., Anagnostopoulou A., Arner A., Montezano A.C. (2018). Vascular smooth muscle contraction in hypertension. Cardiovasc. Res..

[37] Isenberg J.S., Hyodo F., Pappan L.K., Abu-Asab M., Tsokos M., Krishna M.C., Frazier W.A., Roberts D.D. (2007). Blocking thrombospondin-1/CD47 signaling alleviates deleterious effects of aging on tissue responses to ischemia. Arterioscler. Thromb. Vasc. Biol..

[38] Isenberg J.S., Qin Y., Maxhimer J.B., Sipes J.M., Despres D., Schnermann J., Frazier W.A., Roberts D.D. (2009). Thrombospondin-1 and CD47 regulate blood pressure and cardiac responses to vasoactive stress. Matrix Biol..

[39] Frazier E.P., Isenberg J.S., Shiva S., Zhao L., Schlesinger P., Dimitry J., Abu-Asab M.S., Tsokos M., Roberts D.D., Frazier W.A. (2011). Age-dependent regulation of skeletal muscle mitochondria by the thrombospondin-1 receptor CD47. Matrix Biol..

[40] Bauer P.M., Bauer E.M., Rogers N.M., Yao M., Feijoo-Cuaresma M., Pilewski J.M., Champion H.C., Zuckerbraun B.S., Calzada M.J., Isenberg J.S. (2012). Activated CD47 promotes pulmonary arterial hypertension through targeting caveolin-1. Cardiovasc. Res..

[41] Rogers N.M., Sharifi-Sanjani M., Yao M., Ghimire K., Bienes-Martinez R., Mutchler S.M., Knupp H.E., Baust J., Novelli E.M., Ross M. (2017). TSP1-CD47 signaling is upregulated in clinical pulmonary hypertension and contributes to pulmonary arterial vasculopathy and dysfunction. Cardiovasc. Res..

[42] Rogers N.M., Roberts D.D., Isenberg J.S. (2013). Age-associated induction of cell membrane CD47 limits basal and temperature-induced changes in cutaneous blood flow. Ann. Surg..

[43] Nevitt C., McKenzie G., Christian K., Austin J., Hencke S., Hoying J., LeBlanc A. (2016). Physiological levels of thrombospondin-1 decrease NO-dependent vasodilation in coronary microvessels from aged rats. Am. J. Physiol. Heart Circ. Physiol..

[44] Gao Q., Chen K., Gao L., Zheng Y., Yang Y.G. (2016). Thrombospondin-1 signaling through CD47 inhibits cell cycle progression and induces senescence in endothelial cells. Cell Death Dis..

[45] Meijles D.N., Sahoo S., Al Ghouleh I., Amaral J.H., Bienes-Martinez R., Knupp H.E., Attaran S., Sembrat J.C., Nouraie S.M., Rojas M.M. (2017). The matricellular protein TSP1 promotes human and mouse endothelial cell senescence through CD47 and Nox1. Sci. Signal..

[46] Ghimire K., Li Y., Chiba T., Julovi S.M., Li J., Ross M.A., Straub A.C., O’Connell P.J., Rüegg C., Pagano P.J. (2020). CD47 Promotes Age-Associated Deterioration in Angiogenesis, Blood Flow and Glucose Homeostasis. Cells.

[47] Rogers N.M., Yao M., Sembrat J., George M.P., Knupp H., Ross M., Sharifi-Sanjani M., Milosevic J., St Croix C., Rajkumar R. (2013). Cellular, pharmacological, and biophysical evaluation of explanted lungs from a patient with sickle cell disease and severe pulmonary arterial hypertension. Pulm. Circ..

[48] Novelli E.M., Little-Ihrig L., Knupp H.E., Rogers N.M., Yao M., Baust J.J., Meijles D., St Croix C.M., Ross M.A., Pagano P.J. (2019). Vascular TSP1-CD47 signaling promotes sickle cell-associated arterial vasculopathy and pulmonary hypertension in mice. Am. J. Physiol. Lung. Cell. Mol. Physiol..

[49] Wernig G., Chen S.Y., Cui L., Van Neste C., Tsai J.M., Kambham N., Vogel H., Natkunam Y., Gilliland D.G., Nolan G. (2017). Unifying mechanism for different fibrotic diseases. Proc. Natl. Acad. Sci. USA.

[50] Leeming D.J., Genovese F., Sand J.M.B., Rasmussen D.G.K., Christiansen C., Jenkins G., Maher T.M., Vestbo J., Karsdal M.A. (2021). Can biomarkers of extracellular matrix remodelling and wound healing be used to identify high risk patients infected with SARS-CoV-2?: Lessons learned from pulmonary fibrosis. Respir. Res..

[51] Soto-Pantoja D.R., Stein E.V., Rogers N.M., Sharifi-Sanjani M., Isenberg J.S., Roberts D.D. (2013). Therapeutic opportunities for targeting the ubiquitous cell surface receptor CD47. Expert. Opin. Ther. Targets..

[52] Rogers N.M., Ghimire K., Calzada M.J., Isenberg J.S. (2017). Matricellular protein thrombospondin-1 in pulmonary hypertension: Multiple pathways to disease. Cardiovasc. Res..

[53] Cruz Rodriguez J.B., Lange R.A., Mukherjee D. (2020). Gamut of cardiac manifestations and complications of COVID-19: A contemporary review. J. Investig. Med..

[54] Fabrizi F., Alfieri C.M., Cerutti R., Lunghi G., Messa P. (2020). COVID-19 and Acute Kidney Injury: A Systematic Review and Meta-Analysis. Pathogens.

[55] Karmouty-Quintana H., Thandavarayan R.A., Keller S.P., Sahay S., Pandit L.M., Akkanti B. (2020). Emerging Mechanisms of Pulmonary Vasoconstriction in SARS-CoV-2-Induced Acute Respiratory Distress Syndrome (ARDS) and Potential Therapeutic Targets. Int. J. Mol. Sci..

[56] Scutelnic A., Heldner M.R. (2020). Vascular Events, Vascular Disease and Vascular Risk Factors-Strongly Intertwined with COVID-19. Curr. Treat. Options Neurol..

[57] Sanghvi S.K., Schwarzman L.S., Nazir N.T. (2021). Cardiac MRI and Myocardial Injury in COVID-19: Diagnosis, Risk Stratification and Prognosis. Diagnostics.

[58] Maile L.A., Capps B.E., Miller E.C., Aday A.W., Clemmons D.R. (2008). Integrin-associated protein association with SRC homology 2 domain containing tyrosine phosphatase substrate 1 regulates igf-I signaling in vivo. Diabetes.

[59] Allen L.B., Capps B.E., Miller E.C., Clemmons D.R., Maile L.A. (2009). Glucose-oxidized low-density lipoproteins enhance insulin-like growth factor I-stimulated smooth muscle cell proliferation by inhibiting integrin-associated protein cleavage. Endocrinology.

[60] Maile L.A., Allen L.B., Veluvolu U., Capps B.E., Busby W.H., Rowland M., Clemmons D.R. (2009). Identification of compounds that inhibit IGF-I signaling in hyperglycemia. Exp. Diabetes Res..

[61] Maile L.A., Allen L.B., Hanzaker C.F., Gollahon K.A., Dunbar P., Clemmons D.R. (2010). Glucose regulation of thrombospondin and its role in the modulation of smooth muscle cell proliferation. Exp. Diabetes Res..

[62] Maile L.A., Gollahon K., Wai C., Byfield G., Hartnett M.E., Clemmons D. (2012). Disruption of the association of integrin-associated protein (IAP) with tyrosine phosphatase non-receptor type substrate-1 (SHPS)-1 inhibits pathophysiological changes in retinal endothelial function in a rat model of diabetes. Diabetologia.

[63] Abdul-Rahman O., Sasvari-Szekely M., Ver A., Rosta K., Szasz B.K., Kereszturi E., Keszler G. (2012). Altered gene expression profiles in the hippocampus and prefrontal cortex of type 2 diabetic rats. BMC Genom..

[64] Abu El-Asrar A.M., Nawaz M.I., Ola M.S., De Hertogh G., Opdenakker G., Geboes K. (2013). Expression of thrombospondin-2 as a marker in proliferative diabetic retinopathy. Acta Ophthalmol..

[65] Wang J.M., Tao J., Chen D.D., Cai J.J., Irani K., Wang Q., Yuan H., Chen A.F. (2014). MicroRNA miR-27b rescues bone marrow-derived angiogenic cell function and accelerates wound healing in type 2 diabetes mellitus. Arterioscler. Thromb. Vasc. Biol..

[66] Bitar M.S. (2019). Diabetes Impairs Angiogenesis and Induces Endothelial Cell Senescence by Up-Regulating Thrombospondin-CD47-Dependent Signaling. Int. J. Mol. Sci..

[67] Maimaitiyiming H., Norman H., Zhou Q., Wang S. (2015). CD47 deficiency protects mice from diet-induced obesity and improves whole body glucose tolerance and insulin sensitivity. Sci. Rep..

[68] Norman-Burgdolf H., Li D., Sullivan P., Wang S. (2020). CD47 differentially regulates white and brown fat function. Biol. Open..

[69] Cooper D.K.C., Hara H., Iwase H., Yamamoto T., Li Q., Ezzelarab M., Federzoni E., Dandro A., Ayares D. (2019). Justification of specific genetic modifications in pigs for clinical organ xenotransplantation. Xenotransplantation.

[70] Hosny N., Matson A.W., Kumbha R., Steinhoff M., Sushil Rao J., El-Abaseri T.B., Sabek N.A., Mahmoud M.A., Hering B.J., Burlak C. (2021). 3’UTR enhances hCD47 cell surface expression, self-signal function, and reduces ER stress in porcine fibroblasts. Xenotransplantation.

[71] Feng R., Zhao H., Xu J., Shen C. (2020). CD47: The next checkpoint target for cancer immunotherapy. Crit. Rev. Oncol. Hematol..

[72] Oronsky B., Knox S., Cabrales P., Oronsky A., Reid T.R. (2020). Desperate Times, Desperate Measures: The Case for RRx-001 in the Treatment of COVID-19. Semin. Oncol..

[73] Huang F., Yang C., Yu W., Bi Y., Long F., Wang J., Li Y., Jing S. (2016). Hepatitis E virus infection activates signal regulator protein alpha to down-regulate type I interferon. Immunol. Res..

[74] Roquilly A., Jacqueline C., Davieau M., Mollé A., Sadek A., Fourgeux C., Rooze P., Broquet A., Misme-Aucouturier B., Chaumette T. (2020). Alveolar macrophages are epigenetically altered after inflammation, leading to long-term lung immunoparalysis. Nat. Immunol..

[75] Saheb Sharif-Askari N., Saheb Sharif-Askari F., Mdkhana B., Al Heialy S., Alsafar H.S., Hamoudi R., Hamid Q., Halwani R. (2021). Enhanced expression of immune checkpoint receptors during SARS-CoV-2 viral infection. Mol. Ther. Methods. Clin. Dev..

[76] Filbin M.R., Mehta A., Schneider A.M., Kays K.R., Guess J.R., Gentili M., Fenyves B.G., Charland N.C., Gonye A.L.K., Gushterova I. (2021). Longitudinal proteomic analysis of severe COVID-19 reveals survival-associated signatures, tissue-specific cell death, and cell-cell interactions. Cell. Rep. Med..

[77] Boyapati A., Wipperman M.F., Ehmann P.J., Hamon S., Lederer D.J., Waldron A., Flanagan J.J., Karayusuf E., Bhore R., Nivens M.C. (2021). Baseline SARS-CoV-2 Viral Load is Associated With COVID-19 Disease Severity and Clinical Outcomes: Post-Hoc Analyses of a Phase 2/3 Trial. J. Infect. Dis..

[78] Chen P.Z., Bobrovitz N., Premji Z., Koopmans M., Fisman D.N., Gu F.X. (2021). SARS-CoV-2 shedding dynamics across the respiratory tract, sex, and disease severity for adult and pediatric COVID-19. Elife.

[79] Merad M., Martin J.C. (2020). Pathological inflammation in patients with COVID-19: A key role for monocytes and macrophages. Nat. Rev. Immunol..

[80] Mallapaty S. (2021). Kids and COVID: Why young immune systems are still on top. Nature.

[81] Kuo T.C., Chen A., Harrabi O., Sockolosky J.T., Zhang A., Sangalang E., Doyle L.V., Kauder S.E., Fontaine D., Bollini S. (2020). Targeting the myeloid checkpoint receptor SIRPα potentiates innate and adaptive immune responses to promote anti-tumor activity. J. Hematol. Oncol..

