# Supplementary material for: A Potential Role of the CD47/SIRPalpha Axis in COVID-19 Pathogenesis
Source: Curr Issues Mol Biol. 2021 Sep 22;43(3):1212–25. doi: 10.3390/cimb43030086 (PMC8929144; doi:10.3390/cimb43030086)
Supplement: Supplementary file 1 [file cimb-43-00086-s001.zip › cimb-1371614-supplementary-Figures S1-S5.pdf]

Figure S1

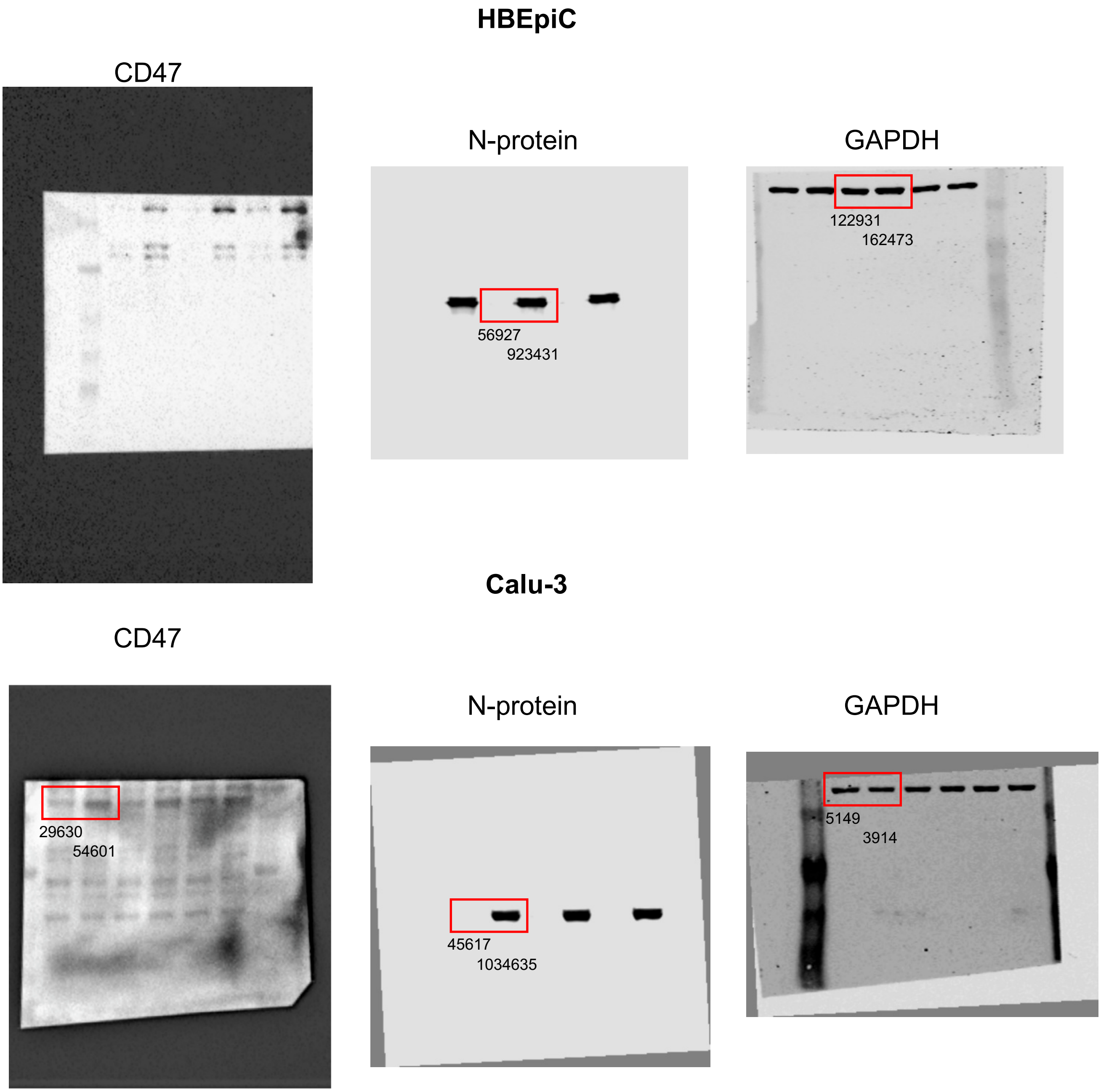

Figure S1. Uncropped Western blots to Figure 1. Bands are indicated by frames. Numbers indicate quantification results.

Figure S2

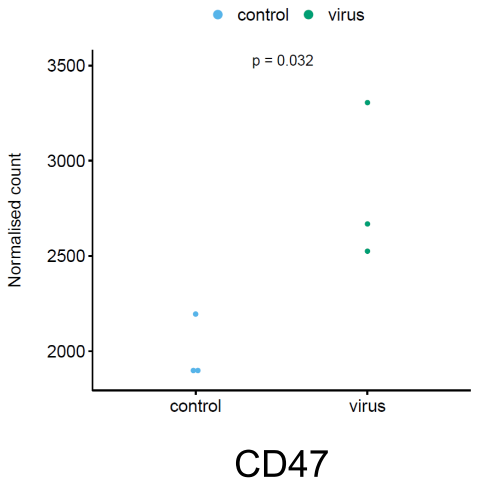

Figure S2. CD47 mRNA levels in SARS-CoV-2-infected Calu-3 cells (data derived from [28]). P-values were determined by two-sided Student’s t-test.

Figure S3

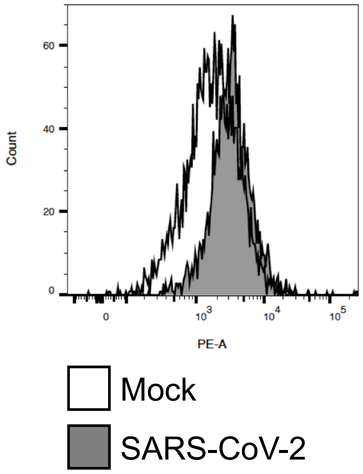

Figure S3. CD47 levels in SARS-CoV-2 (MOI 0.1)-infected Caco2 cells as determined by flow cytometry (FACSCanto II, BD Biosciences). Cells were stained for CD47 using a PE-labelled CD47 antibody (Miltenyi, # 130-123-754, 1:50 dilution) and then fixed with 4% formaldehyde (10 minutes). Isotype REA Control Antibody (S) (human IgG1, PE-labelled, Miltenyi, # 130-113-438, 1:50 dilution) was used as control.

Figure S4

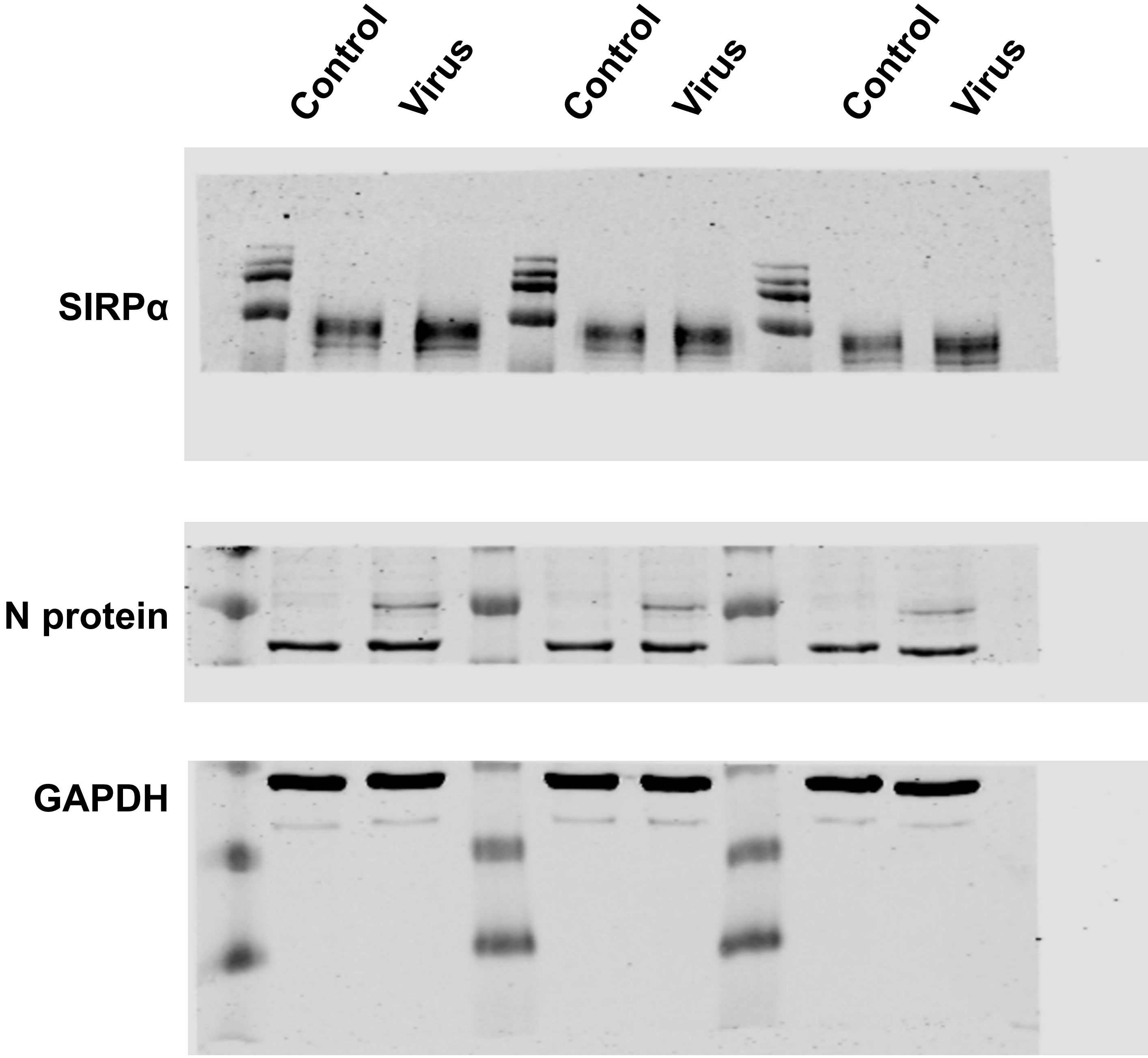

Figure S4. Uncropped Western blots to Figure 2.

Figure S5

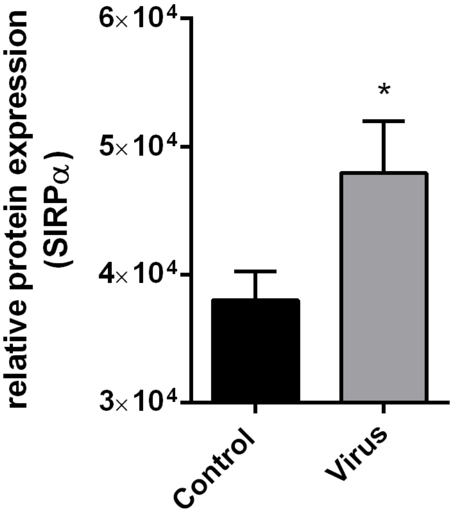

Figure S5. Quantification of SIRPα levels in SARS-CoV-2-infected primary human monocytes. P-values were determined by two-sided Student’s t-test. * $P < 0.05$
